# Assessing risk factors for early hip osteoarthritis in activity-related hip pain: a Delphi study

**DOI:** 10.1136/bmjopen-2015-007609

**Published:** 2015-09-29

**Authors:** K A Jackson, S Glyn-Jones, M E Batt, N K Arden, J L Newton

**Affiliations:** 1 Nuffield Department of Orthopaedics, Rheumatology and Musculoskeletal Sciences, Arthritis Research UK Centre for Sport, Exercise and Osteoarthritis, University of Oxford, Botnar Research Centre, Oxford, UK; 2 Arthritis Research UK Centre for Sport, Exercise and Osteoarthritis, Queen's Medical Centre, Nottingham, UK

**Keywords:** SPORTS MEDICINE, RHEUMATOLOGY, RADIOLOGY & IMAGING

## Abstract

**Objective:**

Hip pain and injury as a result of activity can lead to the development of early hip osteoarthritis (OA) in susceptible individuals. Our understanding of the factors that increase susceptibility continues to evolve. The ability to clearly identify individuals (and cohorts) with activity-related hip pain who are at risk of early hip OA is currently lacking. The purpose of this study was to gain expert consensus on which key clinical measures might help predict the risk of early hip OA in individuals presenting with activity-related hip pain. The agreed measures would constitute a standardised approach to initial clinical assessment to help identify these individuals.

**Methods:**

This Dephi study used online surveys to gain concordance of expert opinion in a structured process of ‘rounds’. In this study, we asked ‘What outcome measures are useful in predicting hip OA in activity-related hip pain?’ The Delphi panel consisted of experts from sport and exercise medicine, orthopaedics, rheumatology, physiotherapy and OA research.

**Results:**

The study identified key clinical measures in the history, examination and investigations (plain anteroposterior radiograph and femoroacetabular impingement views) that the panel agreed would be useful in predicting future risk of hip OA when assessing activity-related hip pain. The panel also agreed that certain investigations and tests (eg, MR angiography) did not currently have a role in routine assessment. There was a lack of consensus regarding the role of MRI, patient-reported outcome measures (PROMs) and certain biomechanical and functional assessments.

**Conclusions:**

We provide a standardised approach to the clinical assessment of patients with activity-related hip pain. Assessment measures rejected by the Delphi panel were newer, more expensive investigations that currently lack evidence. Assessment measures that did not reach consensus include MRI and PROMs. Their role remains ambiguous and would benefit from further research.

**Strengths and limitations of this study**

- This study provides expert consensus on the components of a routine clinical assessment for individuals with activity-related hip pain to help identify groups at risk of future hip osteoarthritis (OA).
- This study provides an overview of current available evidence for hip OA risk factors with summary tables of evidence.
- The literature review was performed as a narrative, not systematic, review.
- The lack of current evidence in young, active populations meant that the expert panel had to extrapolate evidence from studies involving older populations.

## Introduction

The Arthritis Research UK Centre for Sport, Exercise and Osteoarthritis aims to reach a better understanding of the mechanisms linking sport, exercise, injury and osteoarthritis (OA) in order to develop strategies that will enable the whole community to safely and effectively exercise and participate in sport. A standardised approach to assessing patients with activity-related hip joint pain enables future research into identifying cohorts at risk of early hip OA, which then allows meaningful research into prevention and intervention. Currently, there is no general consensus on outcome measures that should be sought when assessing these patients. The aim of this paper is to seek agreement about a standardised approach to assessment from a panel of experts from the fields of OA research, sport and exercise medicine (Sport Advisory Group), physiotherapy, orthopaedic surgery and rheumatology.

The hip joint was identified as a key joint of interest for initial research by the Sports Advisory Group. This group was formed in 2013 of sports medicine experts including the Chief Medical Officers from the national governing bodies of football, rugby, cricket, horse racing, golf, tennis, athletics, dance, Paralympic sport, English Institute of Sport and the Ministry of Defence. The group advises the Arthritis Research UK Centre for Sport, Exercise and Osteoarthritis on key areas for sports-related research.

The burden of symptomatic hip OA is substantial and lifetime risk has been estimated as one in four.^1^ Early hip OA in younger age groups is not insignificant. Prevalence in the 45–54-year age group has been found to be 1 in 20 for symptomatic hip OA and one in five for radiographic hip OA.^2^ The prevalence was found to be slightly higher for men in the younger age groups and higher in women in the over 65s.^2^

Increasing activity levels is a key target for improving the general health of the nation.^3^ ^4^ A potential adverse consequence of activity is joint injury. There is good evidence that traumatic hip joint injury plays an important role in the development of early hip OA.^5^ However, there are other well-recognised factors that influence an individual's risk of OA including non-modifiable factors such as gender, genetics and advancing age^6^ ^7^ and modifiable factors such as obesity and occupation.^5^

In addition to the well-established risk factors, there is evolving evidence of other potentially modifiable factors such as the shape of the femoral head and neck. A focused review of the literature by Harris-Hayes and Royer in 2011 found that an association exists between bony abnormalities found in femoroacetabular impingement (FAI) and acetabular dysplasia and hip OA. Since then, further studies have examined this relationship. A longitudinal cohort study of 455 women showed a 2.7-fold increase in risk of radiographic OA (not symptomatic OA) at 19 years in individuals with a CAM-type deformity at the femoral head/neck junction.^8^ Agricola *et al*^9^ investigated the association between hip shape and clinical OA and total hip replacement (THR) and found that hip shape could not predict clinical OA as defined by the American College of Rheumatism criteria but could predict risk of THR at 5-year follow-up. CAM-type deformities appear to develop in early adolescence and current thinking is that they develop in young individuals exposed to high-impact activity^10^ due to alterations across the growth plate in the hip.^11^ There is growing evidence that FAI predisposes to early onset hip OA. Evidence is not yet clear on the best way to manage FAI. There is a body of opinion that believes that early surgical intervention for treatment of FAI may decelerate the degenerative process in young patients.^12^

Other potentially modifiable risk factors of relevance to an active population is the type of sport or activity participated in and the intensity and volume of participation. These factors have been the focus of a number of systematic reviews and several smaller case–control studies that have found inconsistent results. Several case–control studies have found significantly increased prevalence of hip OA in exprofessional footballers.^13^ ^14^ One study controlled for injury and found a significant increase in hip OA despite the absence of notable hip injuries.^13^ ^14^ Other sports have also been shown to increase the risk of premature hip OA including ice hockey,^15^ handball^16^ and racquet sports.^17^ However, not all the literature is in agreement. A recent systematic review found inconclusive results for the risk of developing hip OA with respect to levels of physical activity or sport specificity in the absence of hip joint injury.^5^

In order to research these modifiable risk factors for hip OA further, an initial step is to be able to accurately identify an at-risk cohort of people who present with activity-related hip pain. This relies on relevant information being obtained as standard at clinical assessment. This may include relevant history, examination, imaging, blood tests and patient-Reported outcome measures (PROMs). The detail of this assessment is not clear from available evidence and there are differences of opinion among specialists.

This study was designed to identify key elements that comprise a routine clinical assessment of a patient with activity-related hip to help predict the risk of early hip OA. This standardisation will enable identification of at-risk cohorts for future research. Since there is a paucity of evidence regarding a minimum standard for assessment, the study used the Delphi process of seeking expert consensus of opinion. The Delphi participants included the Sport Advisory Group and experts from the fields of OA research, sport and exercise medicine, orthopaedics, rheumatology and physiotherapy.

## Methods

A Delphi study is a structured process that invites experts to complete a series of ‘rounds’ (in this study via online surveys) to gather and refine information on the study question, until expert consensus is reached (4).

### Study structure

**Figure 1:**
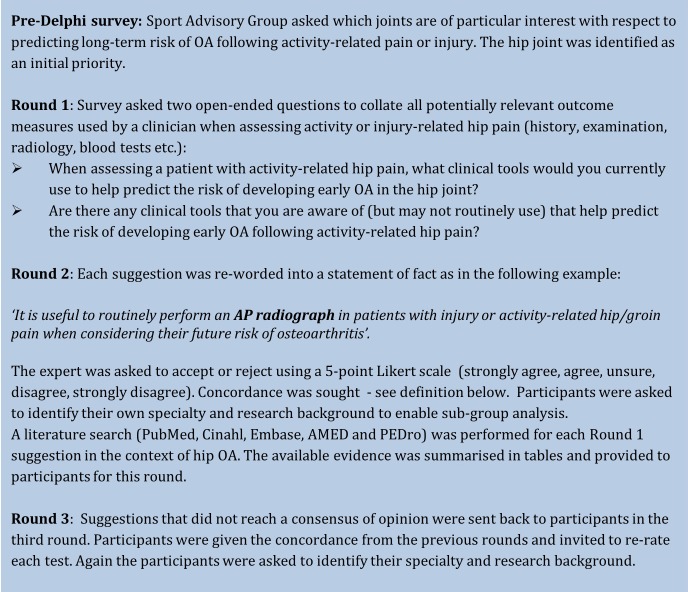
Methodology overview (AP, anteroposterior; OA, osteoarthritis).

The study structure is outlined in figure 1.

### Definition of concordance

Expert consensus was reached for a clinical measure when there was adequate concordance. Concordance was defined as a clinical measure being accepted when ≥60% participants agree and a measure being rejected if ≤20% participants agree. This definition has been used in previous OARSI Delphi studies.^18^ ^19^

### Participant identification

Experts were selected from a wide range of representative bodies relevant to the fields of exercise, sport, sport injuries and OA. These include the Sport Advisory Group (see Introduction section), other sport-specific experts and allied professionals including orthopaedic hip surgeons, rheumatologists, physiotherapists and experts in OA research. The criteria agreed by the authors were the following:

- Chief Medical Officer (or equivalent) of Sporting National Governing bodies
- Ten years clinical experience in relevant specialty (rheumatology, orthopaedics, physiotherapy, sports medicine)
- Researcher who has published in the area of activity-related hip pain or hip OA

An introductory letter and information sheet (Plain Language Statement) were emailed to 33 potential Delphi panel experts and 23 experts responded and participated. One non-clinical researcher declined to participate and there were a further nine non-respondents (5 clinicians, 3 clinical researchers and 1 non-clinical researcher).

The final panel consisted of 23 participants: 12 clinical researchers (3 orthopaedic surgeons, 3 sports medicine physicians, 3 rheumatologists, 3 physiotherapists), 8 clinicians (sports medicine) and 3 non-clinical OA researchers. It was an international panel from the UK, Australia, China, Japan, Sweden and Denmark.

One full-time researcher only completed round 1 and did not provide an identifying email and therefore could not be included in the Delphi study. By the end of the study, one further participant (clinician) had dropped out for unspecified reasons.

### Inclusion/exclusion criteria for participants

All invited experts who completed round 1 and made themselves identifiable to the investigator were included in the study as Delphi participants. The expert panel was selected as detailed above. If the participant did not have access to a computer to complete the online surveys, they were excluded. There were no further exclusion criteria.

### Informed consent

There was no explicit written consent for this study. By completing the round 1 online survey, we assumed there was an implied consent to participate. This was explained to participants in the introductory email.

### Discontinuation/withdrawal of participants from study

Participation in the study was entirely voluntary and withdrawal from the study could occur at any point. The dropout rate was as follows: round 1: 23 participants, round 2: 22 participants and round 3: 21 participants.

### Definition of end of study

The study ended after three rounds of online surveys.

### Literature search

A literature search was performed between September and November 2013 by KAJ on all suggestions from round 1 (see online supplementary file 1). The authors did not identify additional risk factors from their knowledge of the literature or through the search of the current literature. The search was performed on five databases (PubMed, Cinahl, EMBASE, AMED and PEDro). Each literature search used a round 1 suggestion combined with the following core search terms: coxarthrosis, osteoarthritis, arthrosis, hip, risk, predict*.

Each search was performed systematically using the same core search terms on each of the databases listed above. Each study included was rated as per Centre for Evidence-Based Medicine Levels of Evidence guidelines.^20^ This rating was performed by KAJ and reviewed by JLN. This reference was provided to the panel for those not familiar with its use. All studies level 4 and above were included in the evidence tables. In the absence of robust studies in young, active populations, the selection criteria for evidence included risk factors for hip OA in all populations (not restricted by age or activity level). The population characteristics were stated in the evidence tables to allow appropriate interpretation of the study results by the expert panel.

The results of the literature search were summarised in the tables. The tables of evidence were provided to the Delphi participants in round 2 to inform their decision-making process (see online supplementary file 2).

## Results

### Round 1 results

**Figure 2:**
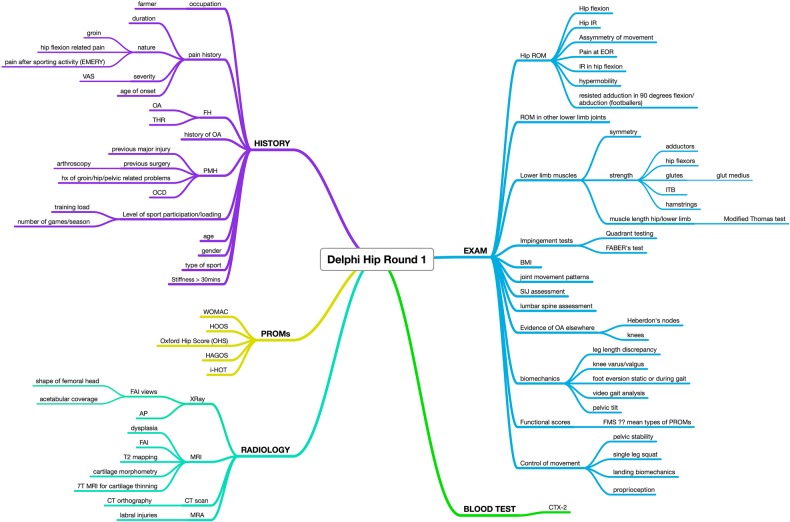
Delphi round 1 results (AP, anteroposterior; BMI, body mass index; EOR, end of range; FABER, flexion abduction and external rotation; FAI, femoroacetabular impingement; FH , family history; FMS, functional movement screen; HAGOS, Copenhagen Hip and Groin Score; HOOS, Hip Disability and Osteoarthritis Outcome Score; i-HOT, International Hip Outcome Tool; ITB, iliotibial band; OA, osteoarthritis; OCD, osteochondral defect; OHS, Oxford Hip Score; PMH, previous medical history; PROMs, patient-reported outcome measures; THR, total hip replacement; WOMAC, Western Ontario McMaster Universities Arthritis Index; VAS, visual analogue scale).

Over 40 suggestions were provided by the Delphi panel in the first round (figure 2). Related suggestions were grouped together for simplicity. The suggestions were categorised into history, examination, blood tests, radiology and PROMs. One suggestion from the surveys was not identifiable as an outcome measure and so thought to be a typing error and had to be omitted.

### Round 2 results

The Delphi participants reached consensus of opinion on 29 statements (table 1): 25 statements were accepted that is, ≥60% agreed or strongly agreed and 4 statements were rejected that is, ≤20% agreed or strongly agreed (table 2). The remaining round 2 statements that did not reach consensus were sent back to the experts in the next round (table 3).

**Table 1:** Accepted suggestions following round 2

Suggestions reaching concordance for acceptance after round 2	Agreed/strongly agreed, %
Radiology	
1. AP radiograph	65
2. FAI views	68
History	
3. Occupation	100
4. Age	91
5. Gender	77
6. Type of sport	91
7. Level of sport participation	95
8. Family history of OA	82
9. Medical history of OA	95
10. Previous hip injury	100
11. Previous hip surgery (eg, arthroscopy)	95
12. Osteochondral defects	81
13. Nature of pain (eg, duration, severity, location)	82
14. History of aggravating movements (eg, flexion)	86
15. Stiffness	71
16. Timing of pain in relation to activity	67
Examination	
17. Absolute range-of-movement of hip	91
18. Pain-related hip movements	83
19. Impingement testing (eg, FADIR or FABER)	83
20. Hypermobility assessment	61
21. Muscle strength around hip and pelvis (eg, hip flexors, gluteal muscles, ITB, hamstrings, adductors)	70
22. BMI	96
23. Lumbar spine assessment	74
24. Evidence of OA elsewhere (eg, Heberden's nodes, knee OA)	83
25. Single leg squat assessment	70

AP, anteroposterior; BMI, body mass index; FABER, flexion abduction and external rotation; FADIR, flexion, adduction, internal rotation; FAI, femoroacetabular impingement; ITB, iliotibial band; OA, osteoarthritis.

**Table 2:** Rejected suggestions following round 2

Suggestions reaching concordance for rejection after round 2	Agreed or strongly agreed, %	Disagreed or strongly disagreed, %
Radiology		
1. CT scan	9	74
2. MRA	9	70
Blood tests		
3. CTX- II	14	36
Examination		
4. Video gait analysis	13	52

MRA, MR angiography.

**Table 3:** Suggestions that did not reach concordance following round 2

Suggestions failing to reach concordance after round 2	Agreed or strongly agreed, %	Disagreed or strongly disagreed, %	Uncertain, %
Radiology			
1. 1.5 T MRI	35	43	22
2. 3 T MRI	48	22	30
3. T2* MAPPING MRI	30	30	40
4. 7 T MRI	22	39	39
Proms			
5. WOMAC	30	48	22
6. OXFORD HIP SCORE	35	35	30
7. HOOS	48	17	35
8. HAGOS	48	9	43
9. i-HOT	35	9	57
History			
10. Age of onset of pain	57	5	33
Examination			
11. Sacroiliac joint assessment	39	30	31
12. Leg length discrepancy	57	22	21
13. Knee varus/valgus	44	26	30
14. Foot eversion	35	35	30
15. Landing biomechanics	30	40	30
16. Proprioception	35	22	43
17. Functional movement control	43	22	35
18. Range of motion of other lower limb joints	48	17	35
19. Symmetry of lower limb muscles	52	17	31
20. Lower limb flexibility/muscle length	43	13	44
21. Pelvic stability	39	17	44

HAGOS, Copenhagen Hip and Groin Score; HOOS, Hip Disability and Osteoarthritis Outcome Score; i-HOT, International Hip Outcome Tool; Proms, patient-reported outcome measures; WOMAC, Western Ontario McMaster Universities Arthritis Index.

### Round 3 (final) results

**Figure 3:**
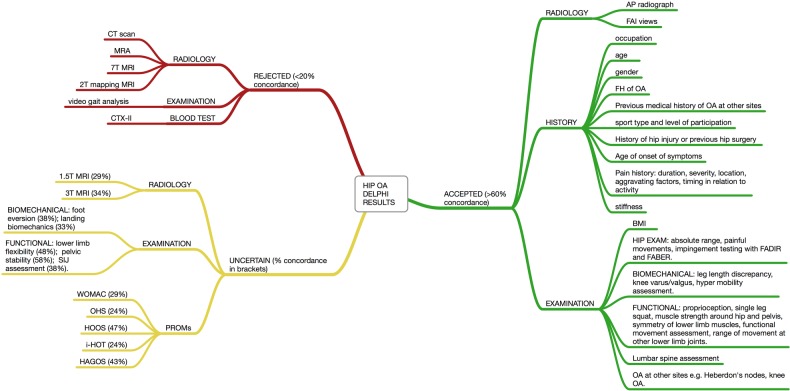
Overview of final Delphi results (AP, anteroposterior; BMI, body mass index; FABER, flexion abduction and external rotation; FADIR, flexion, adduction, internal rotation; FAI, femoroacetabular impingement; HAGOS, Copenhagen Hip and Groin Score; HOOS, Hip Disability and Osteoarthritis Outcome Score; i-HOT, International Hip Outcome Tool; IR, internal rotation; MRA, MR angiography; OA, osteoarthritis; OHS, Oxford Hip Score; PROMs, patient-reported outcome measures; ROM, range of motion; WOMAC, Western Ontario McMaster Universities Arthritis Index).

The Delphi participants reached consensus of opinion on a further nine statements: seven more statements were accepted, two more statements were rejected. Twelve clinical measures failed to reach consensus following the final round. Figure 3 shows an overview of the final results and is divided into clinical measures that were accepted, rejected or failed to reach consensus. The final consensus level is in brackets.

### Analysis of the uncertain suggestions by research background and specialty

The results were broken down for subanalysis by participant's research background and by the participant's specialty. To maintain anonymity, the sole non-clinical researcher was combined with the clinical researcher group. The numbers were too small for meaningful interpretation, but the subanalysis graphs are available (see online supplementary file 3).

## Discussion

The Delphi process has identified, through consensus of opinion, a standardised assessment in the form of history, examination and basic radiographic investigations that the expert panel would routinely perform in individuals with activity-related hip pain to help identify individuals at higher risk of early OA. This assessment is summarised in table 4.

**Table 4:** Overview of agreed standardised assessment following Delphi consensus

History	Examination	Investigations
Age	BMI	AP radiograph hip
Gender	Evidence OA other sites eg, knees	FAI view radiograph hip
Occupation	Leg length discrepancy	
Family history of OA	Knee varus/valgus	
History of hip problems, hip injury or hip surgery	Hypermobility	
History of OA at other sites	Hip absolute ROM/hip painful movement	
Age of onset of symptoms	FADIR impingement test	
Type of sport or exercise	FABER test	
Level of sporting participation	Proprioception	
Pain history (duration, severity, location, aggravating factors, timing in relation to activity)	Single leg squat	
	Lower limb muscle strength and symmetry	
	ROM other lower limb joints	
	Functional movement assessment	
	Lumbar spine assessment	

AP, anteroposterior; BMI, body mass index; FABER, flexion abduction and external rotation; FADIR, flexion, adduction, internal rotation; FAI, femoroacetabular impingement; OA, osteoarthritis; ROM, range of motion.

### History

The agreed points to note in the history include the non-activity-related OA risk factors (eg, family history) as well as factors particular to an individual's sport or exercise. Systematic reviews^5^ ^21^ have established the evidence base for several well-recognised risk factors for hip OA such as previous hip injury, occupations involving heavy lifting and obesity. A large US Defense epidemiological study by Scher *et al*^6^ found increasing age (>40 years) and female gender to be risk factors for hip OA.

The heritability of hip OA has been calculated in twin studies as 50–60% for radiographic OA, independent of environmental or demographic confounding factors.^22^ ^23^ A recent study found that after adjustment for confounders that cause secondary morphological change, individuals with a hereditary predisposition to end-stage hip OA had a higher prevalence of morphological abnormalities associated with hip OA.^24^ Research into the genes responsible is challenging because candidate gene studies and genome-wide association studies show that OA is genetically heterogeneous with each individual common gene variant contributing only modestly to the risk of OA.^22^

Pollard *et al*^24^ found that a family history of end-stage idiopathic OA increases the likelihood of an individual having a CAM deformity with an OR of 2.1 (95% CI 1.3 to 3.5).

There is good evidence that previous joint injury predisposes an individual to developing hip OA.^5^ ^7^ ^25^ The definition of hip injuries varied between studies and included injuries that resulted in lost training time, injuries that resulted in medical consultations or injuries that resulted in fractures or internal derangement of the joint. Cooper *et al*'s study of 611 men and women defined hip injury as the inability to weight bear for at least 1 week and occurring at least 1 year prior to onset of hip pain. In this study, previous hip injury was associated with an overall 4.3-fold increase in the risk of hip OA, greater in men (OR=24.8, 95% CI 3.1 to 199.3) than women (OR=2.8, 95% CI 1.4 to 5.9).^25^ There is also a strong association between congenital hip dysplasia and risk of hip OA.^26^ Perthes disease has been shown to increase risk of subsequent THR.^27^

Level of activity and risk of OA was the subject of a recent systematic review by Richmond *et al*^5^ The review found that joint injury was a clear risk factor for future hip OA, but the findings were inconclusive for level of activity mainly due to the heterogeneous small study designs. However, there are several case–control studies that suggest that an individual who plays sport at the elite level has an increased risk of hip OA even in the absence of hip injury.^13^ ^14^ ^16^ ^28^ In addition, there is evidence that the type of sport played appears to be relevant. The incidence of CAM-type hip morphology is increased in particular sports including football (soccer), basketball and ice hockey.^10^ ^11^ ^29^

### Examination

The standardised examination includes body mass index (BMI), which is known to be a risk factor for lower limb OA (strong association for knee OA, weaker for hip OA) and standard hip range of movement, for which there is evidence that reduced internal rotation is associated with hip OA. The current evidence for BMI as a risk factor for hip OA appears to show a weak, population-based increase in risk. Increased BMI in early and middle adulthood has been shown in one large cohort study^30^ to increase the risk of THR with an HR of 1.29 per 5 kg/m^2^ (95% CI 1.21 to 1.37). Another large cohort study found that the risk of hip OA increased as the BMI increased from an HR of 1.46 if overweight, 1.75 if obese and 1.93 if morbidly obese.^31^ The strength of association between obesity and OA was found to be greater for knee OA than hip OA.^5^ ^30^ Several cohort studies and a case–control study have failed to find a significant association between obesity and risk of hip OA.^32–34^

Restricted hip internal rotation has been shown to be predictive of the presence of hip OA in new presenters to primary care with hip pain.^35^ It may also signify impingement from a CAM deformity as suggested by a number of small studies.^36^ ^37^ Impingement tests have been studied in the context of identifying labral tears or intra-articular pathology. A recent systematic review with meta-analysis concluded that when pretest probability of FAI or labral tear is high, few hip clinical tests actually make a significant change in post-test probability for the potential of FAI/acetabular labral tear existing. Two tests had enough data to support their use as screening tests for FAI or labral tears: FADIR (flexion, adduction, internal rotation) test and the Flex-IR (flexion, internal rotation) test.^38^ Evidence is lacking for the use of any test in the context of predicting early hip OA directly. Biomechanical and functional assessments are included in the routine assessment by consensus of opinion. There is currently a lack of evidence for their use in this context.

One paper was identified regarding self-reported biomechanical abnormalities and risk of hip OA in 1901 men and women.^39^ It found no significant association between knee valgus or varus and hip OA. Leg length inequality was not significantly associated with either hip symptoms or hip OA.^40^ ^41^

### Investigations

Investigations that the panel agreed should be routinely performed include anteroposterior (AP) radiograph of the hip and FAI impingement view radiograph of the hip. AP radiographs may well be considered fairly routine in this context, but FAI view radiographs may not be so widely considered. These views look for FAI by looking at the shape (α angle) of the head/neck junction of the hip. There is currently a lack of uniformity in the literature regarding the cut-off point for the α angle that is considered ‘normal’. Radiological assessment of CAM deformity (also known as CAM lesion or pistol grip deformity) has been increasingly studied as a potentially relevant predictor of OA risk. Several cohort studies of non-elite populations have performed radiographic assessment of a CAM deformity through α-angle measurement. The α angle of Nötzli^42^ estimates the degree at which the radius of curvature of the femoral head begins to increase.^43^ The definition of a CAM deformity differs between studies varying from an α angle >50°^44^ ^45^ to an α angle >65°.^8^ ^46–48^ A recent study has tried to address this uncertainty by assessing the distribution of α angles in 2005 men and women aged 45–65 years from two large cohorts. The resulting distribution was used to determine a threshold of 60° for presence of a CAM deformity.^49^

A cohort study by Thomas *et al*^8^ found that a CAM deformity defined as an α angle >65° on an AP radiograph was associated with a 2.7-fold increased risk of radiographic OA in women (95% CI 1.63 to 4.33, p<0.001). A nested case–control study by Thomas *et al*^47^ found that a CAM deformity defined as an α angle >65° was associated with a sixfold increase in the risk of total hip arthroplasty in women (95% CI 2.04 to 17.59, p<0.001). A cross-sectional cohort study by Gosvig *et al*^50^ found that a pistol-grip deformity (CAM deformity) was associated with a risk ratio for developing hip OA of 2.2 (95% CI 1.7 to 2.8).

Other smaller studies found that having a CAM deformity of the hip is associated with an increased risk of subsequent hip OA,^51^ ^52^ a fourfold risk (OR 4.0, 95% CI 1.26 to 12.71) of acetabular cartilage damage^48^ or an increased risk of THR.^9^

### Rejected assessment measures

The six rejected suggestions included newer, more sophisticated imaging, video gait analysis and CTX-II blood test. These procedures are costly or invasive or both. There is no current evidence to support their use in the context of routine assessment.

MRI is evolving with new technology allowing greater detail (eg, 7 T MRI) and increased information regarding damaged cartilage (eg, functional MRI). Functional MRI such as delayed gadolinium-enhanced MRI is being used to demonstrate cartilage damage. Normal cartilage has a high glycosaminoglycan (GAG) content and damaged cartilage a low GAG content. The uptake of gadolinium is inversely proportional to the GAG content of the cartilage, so damaged cartilage will take up a higher concentration. Although the relationship between cartilage damage and OA is not fully understood, there have been several small or preliminary studies looking at the potential for functional MRI to be used as radiological biomarkers for early hip OA.^53–59^

The only blood test defined by the expert group was serum CTX-II. The literature search did not identify any evidence for serum CTX-II as a potential predictor of hip OA. The ECHODIAH cohort was a 3-year longitudinal multicentre trial that identified urinary (not serum) CTX-II as a potential predictor of structural progression of hip OA.^60^ The patients in the study already had established hip OA and were in the age group 50–75 years.

CTX-II is one of a number of potential wet biomarkers that has been researched with the hope of providing a diagnostic tool. The majority of OA wet biomarker studies have looked at knee OA, not hip OA. Recent editorials and reviews of wet biomarkers for OA prediction highlight their current poor sensitivity and specificity and, as a result, are currently still research tools.^61–63^

### Measures that failed to reach consensus

PROMs are useful in clinical and research settings. However, they are often very detailed which precludes routine clinical use. To address this, there are attempts to provide validated shorter versions of some PROMs (eg, i-HOT 33 and the shorter i-HOT 12). None of the PROMs identified are currently validated for use as predictive tools for the future hip OA risk in active people. The Western Ontario McMaster Universities Arthritis Index (WOMAC), Hip Disability and Osteoarthritis Outcome Score (HOOS) and Oxford Hip Score (OHS) were developed and validated to monitor hip OA symptoms^64^, hip disability symptoms^65^ and to assess outcome after hip surgery,^66^ ^67^ respectively. The Copenhagen Hip and Groin Score (HAGOS) and the International Hip Outcome Tool (i-HOT) have been developed more recently to monitor hip and groin symptoms in young active populations^68–70^ and, as such, may prove useful for researching risk of future hip OA.

The panel could not agree on the role of 1.5 T and 3 T MRI. MRI can identify abnormal hip morphology and pathology. Its role in identifying early hip OA is unclear. There is no available evidence that it is superior to plain radiographic FAI views for identifying CAM lesions, and therefore its comparative expense prevents it being a first-line investigation of choice for this purpose.^71^ More research is needed to prove that the additional information is useful and cost-effective in routine clinical practice.

## Conclusion

This Delphi study provides a standardised approach to the assessment of patients with activity-related hip. The final agreed assessment is summarised in table 4.

Assessment measures rejected by the Delphi panel were newer, more expensive investigations that currently lack evidence. Those that did not reach consensus include MRI and PROMs. Their role remains ambiguous and would benefit from further research (box 1).

Box 1 **Priorities for future research**

- A need to develop prospective cohorts of young, active people with hip pain.
- Research to identify and validate a patient-reported outcome measures that can be used in this population to help identify and monitor those at higher risk of early hip osteoarthritis.
- Further research in imaging techniques to identify optimal investigations for patients with activity-related hip pain.
